# Moist Biogas Conversion in a Plasma–Catalytic System

**DOI:** 10.1021/acsomega.1c05350

**Published:** 2021-12-06

**Authors:** Michał Młotek, Michalina Perron, Katarzyna Tutaj, Krzysztof Krawczyk

**Affiliations:** Warsaw University of Technology, Noakowskiego 3, 00-664 Warszawa, Poland

## Abstract

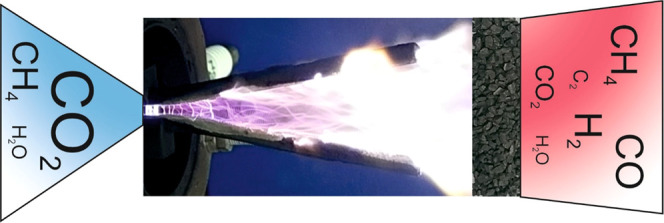

The limited resources of conventional fuels and their negative impact on the environment require scientists to search for alternative energy sources. One of the promising renewable sources of energy is biomass. The energy stored in biomass can be used in various ways. It can be combusted, gasified, or fermented, which leads to obtaining biogas. The main components of biogas are carbon dioxide and methane. The aim of this study was to convert in plasma and plasma–catalytic systems low methane biogas into a hydrogen and carbon monoxide mixture, which will allow for a wider range of potential applications. The combustible gas content increased in both systems. The effect of the water vapor content was investigated. It affects the conversion of CH_4_ and CO_2_ and significantly reduces soot formation (calculated by the carbon balance). It was possible to increase the content of flammable gases by about 20%. The highest molar fraction, 0.16, of hydrogen was obtained with the reduced cobalt catalyst.

## Introduction

The limited resources of conventional fuels and their negative impact on the environment require scientists to search for alternative energy sources. One of the promising renewable sources of energy is biomass. The energy stored in biomass can be used in various ways. It can be combusted, gasified, or fermented, which leads to obtaining biogas.^1^ Fermentation is widely used to produce biogas from different types of waste: the organic fractions of household waste, industrial waste, brewery waste, sewage sludge, and herbaceous feedstocks. The main components of biogas are carbon dioxide and methane.

Problems with the use of biogas are a high carbon dioxide concentration, even up to 70 mol %, a low calorific value of 20.8–23.6 MJ/m^3^, and the sulfur and moisture content. The moisture content of biogas is very high and may cause condensation of water in pipelines whose subsequent reactions with acidic gases contained in biogas lead to corrosion.^2^ For these reasons, biogas cannot be used directly after synthesis as fuel for internal combustion engines. It is necessary to purify^3,4^ and convert biogas into a combustible gas.^3,5,6^ Therefore, new methods of modifying the composition of biogas are sought, a method that will increase its calorific value and allow using it to generate electricity, heat, or use as engine fuel. Biogas composition is not constant, so the method of biogas processing must be effective over a wide range of methane and carbon dioxide concentrations.

There are two ways to upgrade biogas: the first is by removing as much CO_2_ as possible. For this purpose, cryogenic, membrane, and biological methods can be used. After CO_2_ removal, the biogas can be used as fuel in internal combustion engines or transferred to a natural gas pipeline. The second is processing it to increase the concentration of combustible components. Thermal,^7^ catalytic,^8^ and plasma^9,10^ methods are used for this purpose. Ni-containing carrier catalysts have been most commonly used.^7,11^ However, at temperatures below 300 °C, a reaction does not occur. In the temperature range 300–550 °C, the reaction rate of methane and carbon dioxide is very low, even in active Ni-containing catalysts.^8,11^ Moreover, it was found that the addition of hydrogen into gaseous fuels increases combustion stability and speed.^12−14^

In addition, biogas can be used by processing it to higher hydrocarbons,^15−17^ oxygenates,^18,19^ or synthesis gas,^11,20^ which can be a raw feedstock for green ammonia or methanol. This requires the processing of biogas to contain hydrogen and carbon monoxide. Such reactions can be carried out in catalytic,^21−23^ plasma,^8,24^ and plasma–catalytic^25,26^ processes.

Known catalytic methods for converting a methane and carbon dioxide mixture to syngas (reaction 1) are energy-intensive and not used on an industrial scale.
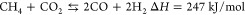
1A review of the methane conversion with CO_2_ in hybrid plasma–catalytic systems was given by Istadi^27^ and Puliyalil.^28^ Various nonthermal plasma methods have been used to convert CO_2_ in different kinds of discharges: RF, DBD, GD,^10,17−19,25−27^ and various pressures. The interest in the conversion of CO_2_ to CO and O_2_ has increased as CO is a chemical feedstock for the synthesis of a range of chemicals. However, dissociation of CO_2_ is a high-energy-consumption reaction (reaction 2).^29^ Gliding discharge is considered a method for CO_2_ dissociation, with energy efficiency in the range of 25–30% and conversion limited to 8–9%.^30^
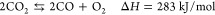
2The application of plasma–catalytic methods to enrich in hydrogen low methane gas diluted with carbon dioxide is the subject of research in many scientific centers.^2,8,19,27−29,31−33^ The aim of this study was to convert moist and low methane biogas into a hydrogen and carbon monoxide mixture, which will allow for a wider range of potential applications. A similar outlet gas composition was received by Xia et al.^24^

## Results

**Figure 1:**
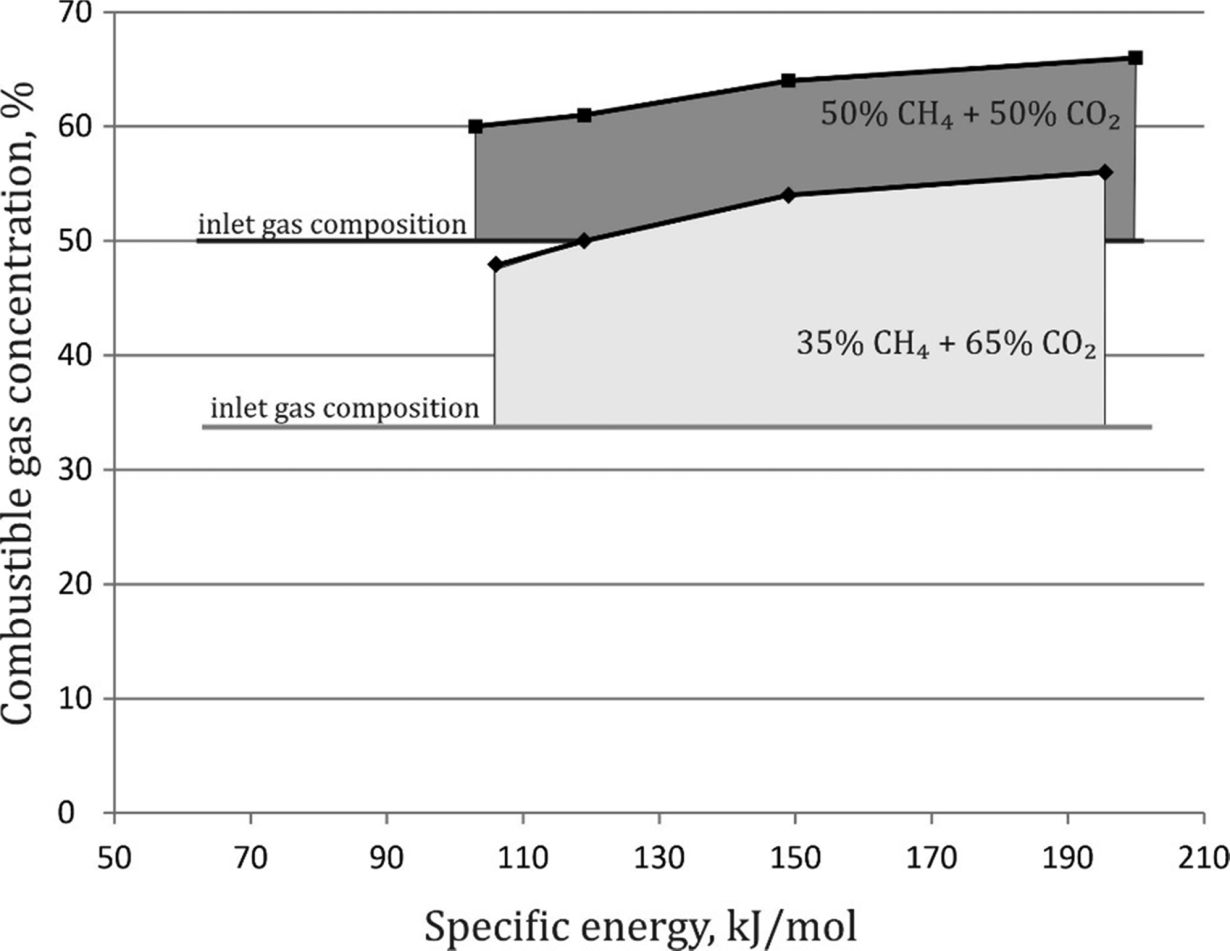
Effect of specific energy and the gas composition on the concentration of combustible gases. The temperature was in the range of 250–380 °C.

The methane and carbon dioxide conversion products formed during this process are hydrogen, carbon monoxide, C_2_ hydrocarbons, and soot (calculated by the carbon balance). The GC-MS analysis did not detect any higher aliphatic or aromatic hydrocarbons in the reaction products. The composition of the products depends on the process conditions, such as the initial concentrations of biogas components, the discharge power, and the gas moistness. The gas containing 35 and 50% methane was converted to be used as a fuel in internal combustion engines. The use of gliding arc plasma increases the combustible gas concentration. For both initial methane concentrations of 50 and 35%, the combustible gas content was increased. The increase in the concentration of combustible components increases with the increase in specific energy (the plasma power normalized by the gas flow rate). It is higher for gas with a lower methane content (35%). A higher concentration of flammable gases was obtained using gas with the initial composition of 50% CH_4_ + 50% CO_2_ in the entire range of the specific energy used. In the case of a concentration of 35%, the amount of combustible components has been increased to such an extent that the gas obtained after the process is combustible (Figure 1). The problem of running the process under such conditions is the formed soot, which is an undesirable byproduct.

### Effect of Water Vapor Addition

Water vapor was added to the converted gas to overcome the problem of soot formation. Introducing water vapor to the mixture of 35% CH_4_ + 65% CO_2_ processed in the gliding discharge reduces methane and carbon dioxide conversion. The water vapor content affects the conversion of methane more than carbon dioxide. A reduction in methane conversion and a slight decrease in CO_2_ conversion result in the increase in the content of combustible gases in the gas after the process it allows to use as a fuel. The advantage of carrying out the process under such conditions is a significant reduction in soot formation, which would have to be systematically removed from the reactor. The example of the carbon balance and soot formation is given in Table 1.

**Table 1:** Effect of Water Vapor on Soot Formation Calculated from the Carbon Balance^a^

	without water vapor	with water vapor
gas flow rate [Nl/h]	450	
discharge power [W]	570	556
total carbon introduced [mol/h]	20.09	20.02
total carbon in outlet gas [mol/h]	19.10	20.01
ΔC [mol/h]	0.99	0.01
*XW*_0_[C] → Cb	0.05	0.0005
total hydrogen introduced [mol/h]	25.90	24.97
total hydrogen in outlet gas [mol/h]	26.02	25.03

a ΔC—difference in the carbon balance and *XW*_0_[C] → Cb—overall entered carbon converted into soot.

**Figure 2:**
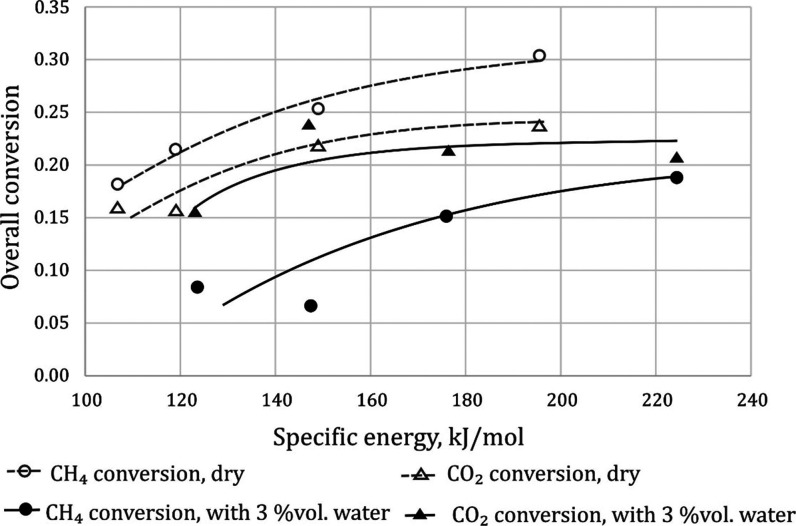
Effect of specific energy and water vapor on the conversion of CO_2_ and CH_4_. Gas composition: 35% CH_4_ + 65% CO_2_. The temperature was in the range of 250–380 °C.

The addition of steam reduces the amount of CO and H_2_ formed compared to the dry gas process. The water vapor slightly reduced the amount of hydrogen obtained. In the steam-containing gas, higher specific energy was obtained. Therefore, the maximum molar fraction of hydrogen in the gas discharged from the reactor is similar in the dry and moist gas, namely, 0.14 and 0.12, respectively (Figure 2).

**Figure 3:**
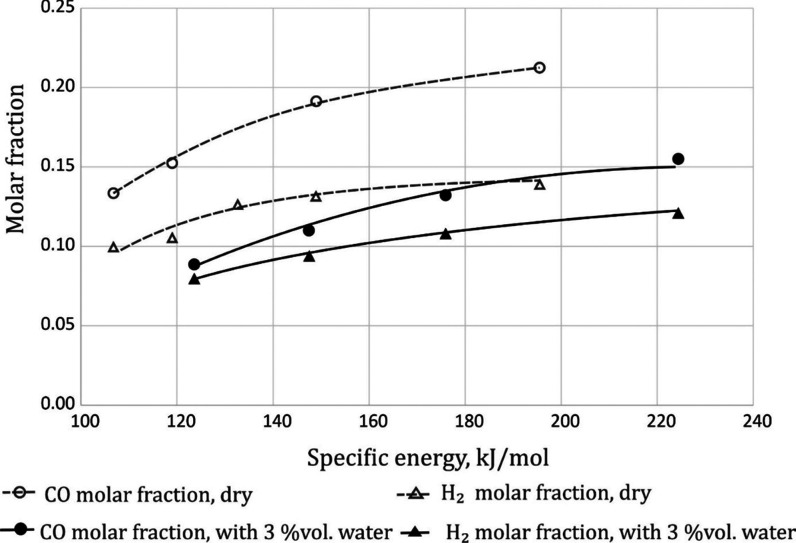
Effect of specific energy on the molar fraction of CO and H_2_. Gas composition 35% CH_4_ + 65% CO_2_. The temperature was in the range of 250–380 °C.

On the other hand, the carbon monoxide content significantly differs as a result of the introduction of water vapor. In dry gas, its content is about 30% higher than in moist gas. The highest molar fraction of carbon monoxide in the gas after the reaction was 0.21 (Figure 3).

**Figure 4:**
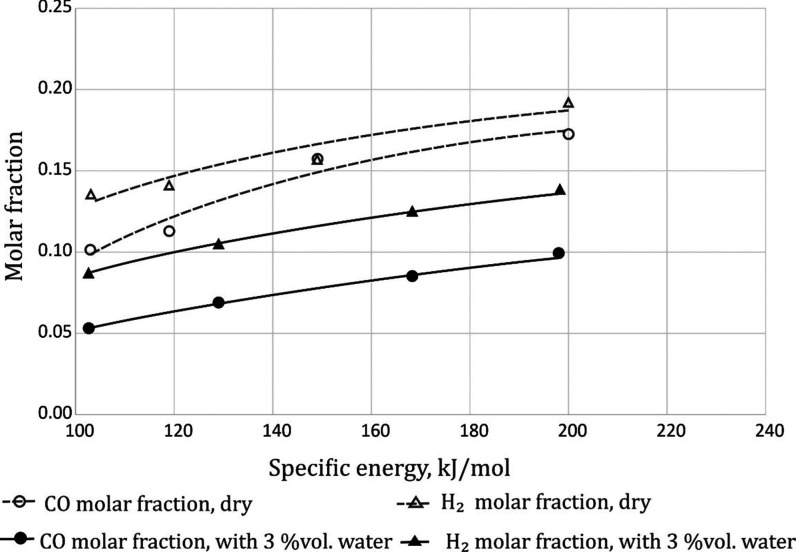
Effect of specific energy on the molar fraction of CO and H_2_. Gas composition 50% CH_4_ + 50% CO_2_. The temperature was in the range of 250–380 °C.

Using the gas containing 50% CH_4_ + 50% CO_2_ similarly as in the case of 35% CH_4_ + 65% CO_2_, higher molar fractions of CO and H_2_ were obtained using the gas without the addition of water vapor (Figure 4). The molar fraction of hydrogen, regardless of whether it is in dry or moist gas, is higher for 50% CH_4_ than for 35% CH_4_. This is due to a higher initial methane concentration.

In contrast, a higher concentration of carbon monoxide was obtained in the postreaction gases in the case of the stream with the initial composition of 35% CH_4_ + 65% CO_2_. For this concentration, the process significantly increases the content of combustible gases. The gas processed by this method, whether with the addition of steam or not, can be used as a fuel for internal combustion engines.

### Influence of the Catalytic Bed

It was verified whether gases with a low methane content could be processed in the same way in the plasma–catalytic system. The effect of Co, Mn, and Ni catalysts on methane conversion was investigated. The methane concentrations were 20 and 30%.

**Figure 5:**
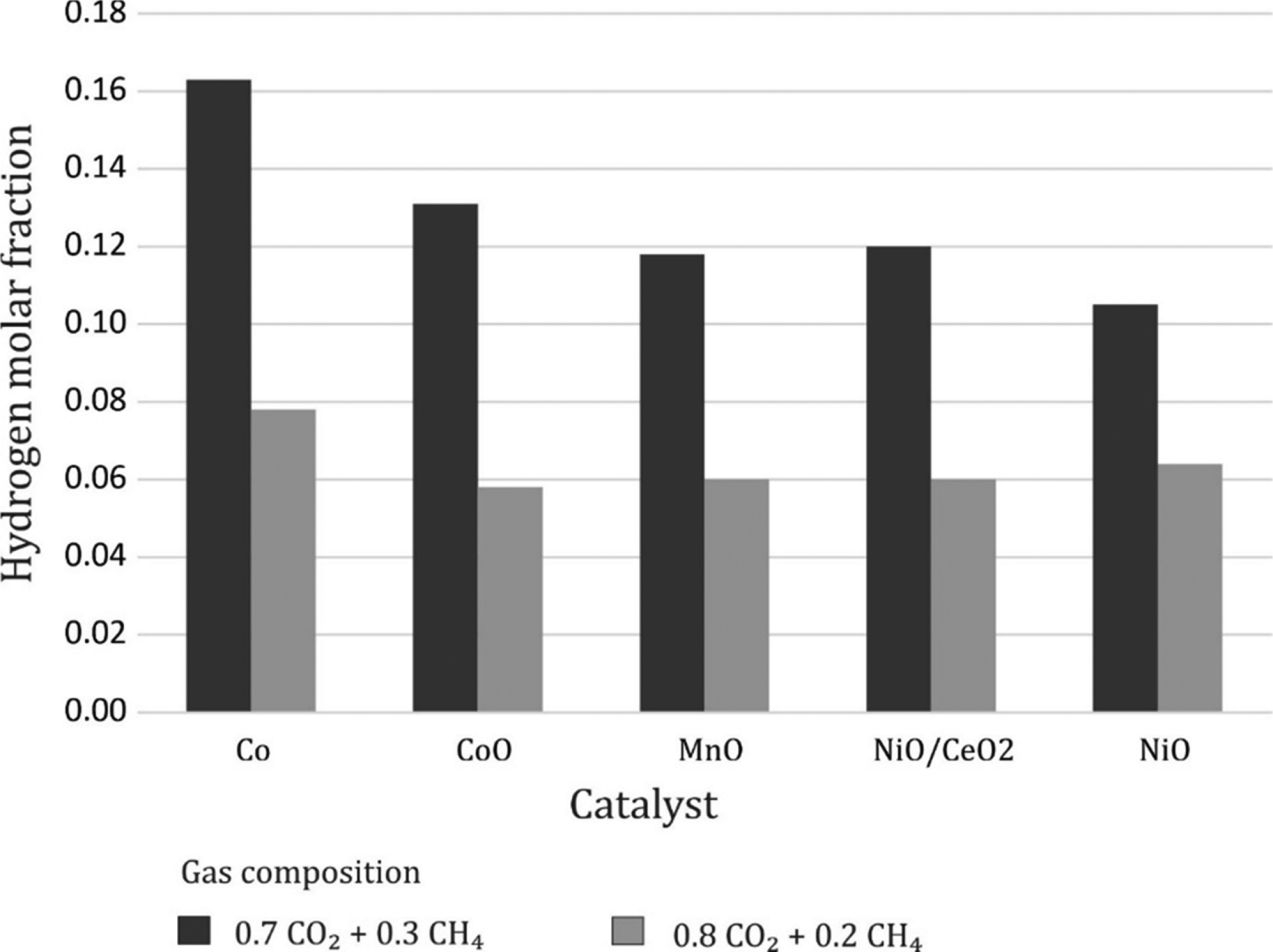
Effect of catalysts on hydrogen concentration. Discharge power ∼550 W, gas flow rate 375 Nl/h, and specific energy ∼120 kJ/mol. The temperature was in the range of 410–440 °C.

The influence of the catalysts on the molar fraction of hydrogen and carbon monoxide in the gas containing 30% CH_4_ was observed. For this concentration, it was possible to increase the content of flammable gases by about 15%. Cobalt catalysts and cesium-promoted nickel catalysts showed the highest activity (Figure 5). The molar fraction of hydrogen obtained with the reduced cobalt catalyst was over 0.16. It was the highest value of the mole fraction of hydrogen in a gas containing water vapor. The conversion of methane and carbon dioxide under these conditions was 34 and 38%, respectively.

When using gas with an initial methane concentration of 20%, no significant effect of the catalytic bed was observed on the hydrogen content of the gas after the reaction. For this concentration, it was possible to increase the content of flammable gases to about 35%. Despite the fact that such gas cannot be used as a fuel, an interesting result is the conversion of CO_2_, which was 20%.

**Figure 6:**
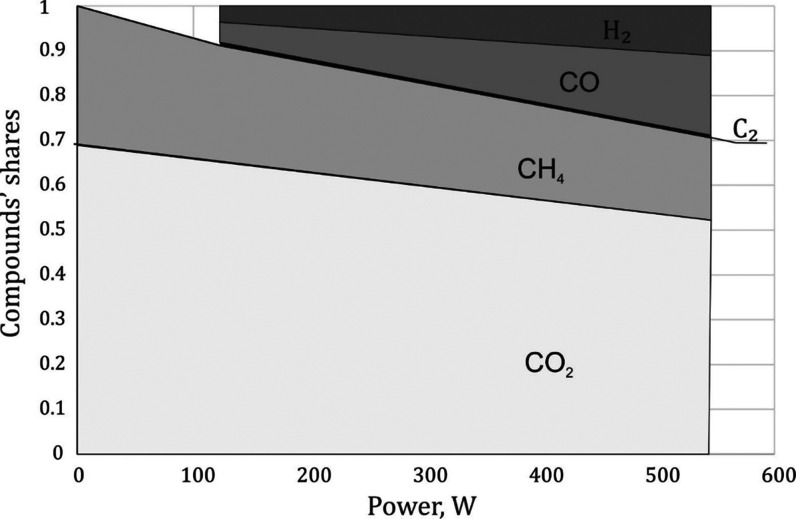
Effect of discharge power on the outlet gas composition with a reduced cobalt catalyst. Gas composition 30% CH_4_ + 70% CO_2_ and overall gas flow rate 450 Nl/h. The temperature was in the range of 200–440 °C.

The calculations were made for the gas with the initial composition of 30% CH_4_ + 70% CO_2_. The detailed composition of the gas after the process is shown in Figure 6. A mass balance was performed, and on its basis, the molar fractions of individual components of the postreaction mixture were calculated. Compounds’ shares are the proportions of individual gas components arranged in order: CO_2_, CH_4_, C_2_, CO, and H_2_. After the process, the new compounds were formed: carbon monoxide, hydrogen, and trace amounts of C_2_ hydrocarbons improving gas quality. Carbon dioxide and methane concentrations decreased during the reaction, from an initial 70 to 51% and from 30 to 18%, respectively. The amount of hydrogen and carbon monoxide increased with increasing discharge power. However, the amount of C_2_ hydrocarbons remained unchanged. It may indicate that soot was formed in trace amounts under the process conditions because the synthesis of unsaturated C_2_ hydrocarbons is an intermediate stage of soot formation.^34^

## Discussion

Gliding discharge plasma can be used to modify the composition of biogas in a wide range of initial concentrations of methane and carbon dioxide. The composition of the products depends on the process conditions, such as the initial concentrations of biogas components, the discharge power, and the gas moistness. In gas processing without the addition of water, soot appears in the reaction products even at low methane concentrations,^35,36^ which may cause discharge failure or poisoning of the catalyst when plasma–catalytic systems are used. The ChemCAD calculated equilibrium conversion of gas mixture 0.35 CH_4_ + 0.65 CO_2_ into soot was 0.66–0.61 in the temperature range of 200–400 °C. The equilibrium conversion of methane and CO_2_ in this temperature was above 0.93 and 0.61, respectively. It allows concluding that the plasma process is conducted far from a state of equilibrium. However, the kinetic issue influences soot and hydrogen formation.^37^ One commonly known method of limiting carbon black formation is the addition of steam. Under such conditions, both reactions, methane reforming and conversion of the formed carbon, can occur. These reactions are very advantageous because of the significant reduction in the emission of soot and the formation of additional amounts of hydrogen and carbon monoxide, which increases the content of combustible gases in the processed gas.
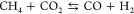
3
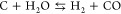
4The disadvantage of running the process in moist gas is the significant reduction of the methane conversion at each applied initial concentration. This may be due to a change in the electrical conditions of the plasma generated in the moistness gas or the methanation reaction of carbon monoxide.
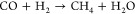
5However, in the case of carbon dioxide, a slight influence of the water vapor content on its conversion degree with the use of gas with a concentration of 65% CO_2_ may be the result of CO conversion with water vapor. This is a reaction that produces extra hydrogen and consumes carbon monoxide.
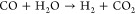
6The entire process carried out is a radical process. Many radicals can be generated in a plasma in a gas containing CH_4_, CO_2_, and H_2_O, e.g., methylene, hydrogen, hydroxyl, and oxide radicals.^38^
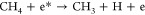
7
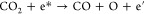
8
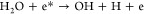
9The radicals can react and recombine in a variety of ways. As a result, the gas composition after the process may vary depending on the initial concentration of individual substrates.^38^ The effect of these reactions may be the regeneration of substrates, which is associated with a significant reduction in energy efficiency or the formation of undesirable products, e.g., soot or acetylene. The mechanism of soot and acetylene formation is shown in reactions 10–14, and in the gliding discharge, the high methane content in the processed gas favors the course of these reactions.
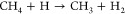
10
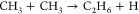
11
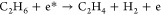
12
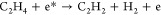
13
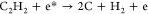
14In the plasma of gliding discharge, reactions 10–13 are very fast, so the product of methane conversion at a high methane concentration is soot and acetylene.^39−41^ This causes a reduction in the plasma selectivity of methane conversion into hydrogen and carbon monoxide. The selectivity of plasma processes can be improved using an active catalyst in the plasma–catalytic system. This requires heating the catalyst to the appropriate temperature or activating the reagents on its surface. Using a plasma–catalytic system increases the reaction rate in which hydrogen and carbon monoxide are formed or reduces the methane coupling reaction. In this paper, the most active in the biogas conversion process was the reduced cobalt catalyst. A high hydrogen molar fraction was obtained at a 30% CH_4_ concentration. The high degree of conversion of methane and carbon dioxide obtained under such conditions may be the effect of increasing the rate of the CH_4_ reaction with CO_2_.

## Conclusions

Biogas with low methane concentration can be processed to improve its quality in the plasma and plasma–catalytic system. After the modification, it can be used as fuel for internal combustion engines. The main products of the reaction were hydrogen, carbon monoxide, and traces of C_2_ hydrocarbons. Aromatic hydrocarbons or oxygenates have not been found in the outlet gas. The soot formation was reduced when the process was conducted in moist gas. The increase of specific energy increases the conversion of the substrates and the molar fractions of hydrogen and carbon monoxide. Cobalt and cerium-promoted nickel catalysts have the highest activity during the conversion of biogas to hydrogen and carbon monoxide.

## Experimental Section

The experiments were conducted in a plasma and plasma–catalytic system with a two-electrode gliding discharge reactor. In plasma–catalytic studies, the catalyst bed was placed above the end of the electrodes. The reactor was powered by an alternating current with a frequency of 100 Hz. The discharge power was regulated with an ENDA thyristor power regulator. Methane and carbon dioxide gases of 99.99% purity were dosed by mass flow controllers (MFC1 and MFC2).

**Figure 7:**
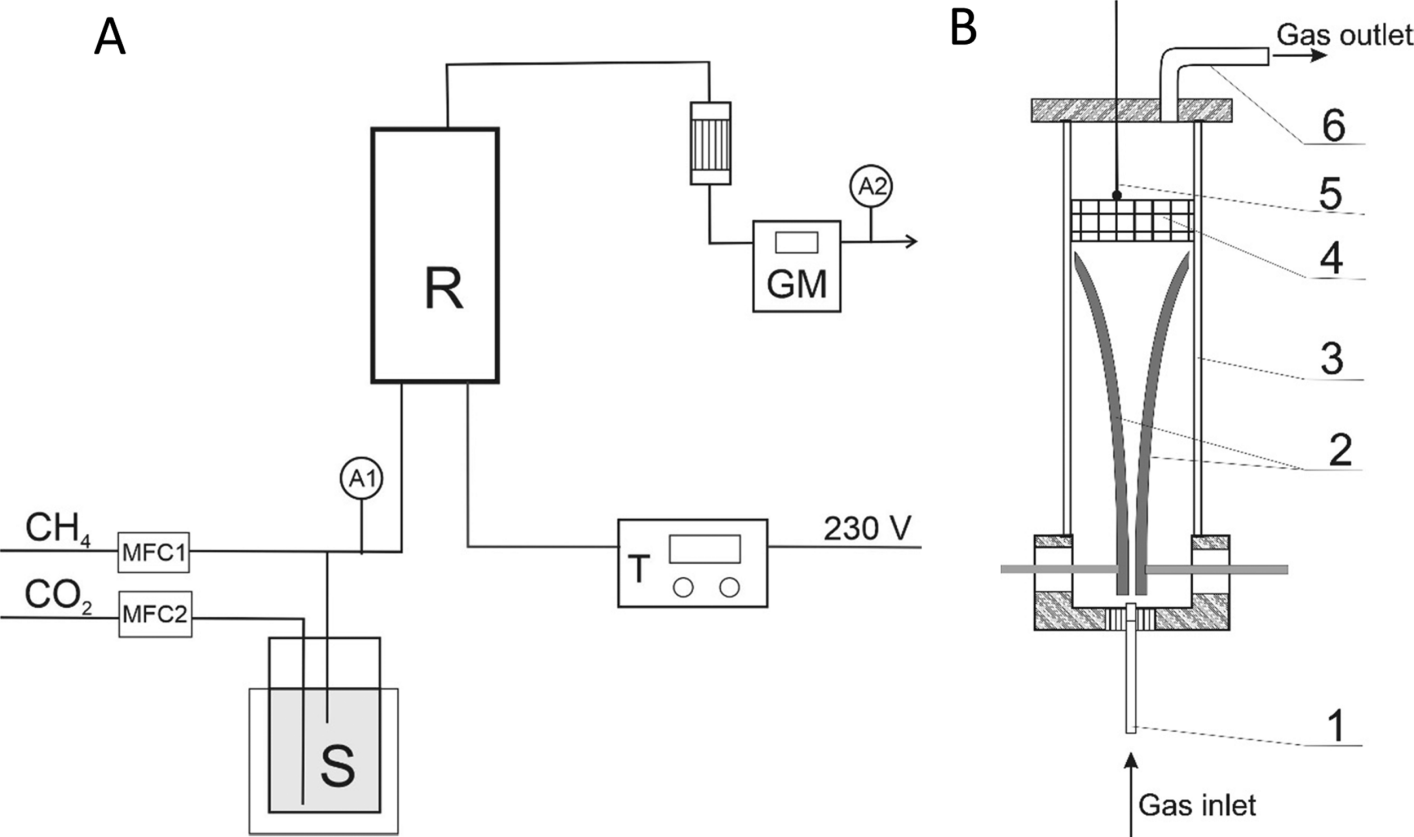
(A) Experimental setup: R—reactor, S—scrubber with water, T—power supply, GM—gas counter, MFC1, MFC2—mass flow controllers for CH_4_ and CO_2_, respectively, and A1 and A2—gas sample collection points. (B) Reactor scheme: 1—gas inlet, 2—electrodes, 3—quartz tube, 4—catalysts bed, 5—thermocouple, and 6—gas outlet.

**Figure 8:**
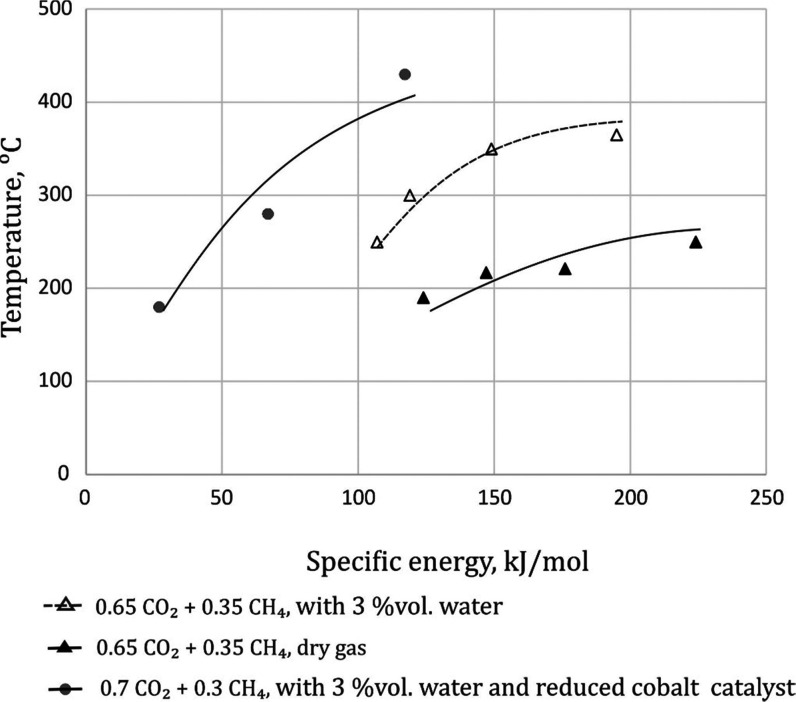
Effect of specific energy, gas composition, and catalyst bed on the temperature of the process.

Upstream of the inlet to the reactor (R), carbon dioxide was directed to a scrubber with water at 80 °C (P). After saturating carbon dioxide with water vapor, it was mixed with methane and directed to the reactor. After cooling, the gas flow was measured with a gas meter (GM) (Figure 7A). The tests were carried out at atmospheric pressure using a gas flow rate in the range of 375–450 Nl/h and an initial CO_2_ concentration of 50–80 mol %. The outlet gas temperature depended on specific energy, gas composition, and catalyst bed presence (Figure 8). The range of process temperature was 200–440 °C.

The gliding discharge reactor consisted of a quartz tube with an internal diameter of 39 mm, a nozzle with a diameter of 0.9 mm, and two electrodes with a length of 70 mm (Figure 7B). The reactor was positioned vertically. Gases were introduced into the reactor through the bottom of the reactor. The distance between the electrodes increased with height. A thermocouple and a postreaction gas outlet were introduced through the upper part of the reactor. In a plasma–catalytic system, a fixed bed of a solid catalyst with 25 mm height was placed 7 mm above the discharge zone.

### Definitions


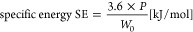
15
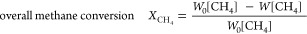
16
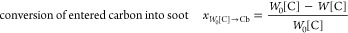
17
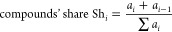
18

*P*—discharge power [W], *W*_0_—overall inlet gas flow rate [mol/h], *W*_0_[C] and *W*[C]—total moles of carbon at the inlet or the outlet of the reactor, respectively [mol/h], *W*_0_[CH_4_] and *W*[CH_4_]—methane flow rates at the inlet or outlet, respectively [mol/h], *W*_0_[CO_2_] and *W*[CO_2_]—carbon dioxide flow rates at the inlet or outlet, respectively [mol/h], and *a_i_*—molar fraction of single gas.

### Catalyst Preparation

Four catalytic systems containing metal oxides deposited on a ceramic support with Al_2_O_3_ have been developed. The grain size of the catalyst was in the range of 1.6–3 mm. The metal content by weight of the catalyst was 10 wt %. The catalysts were obtained by dry impregnating the Al_2_O_3_ support with solutions of nitrates, the appropriate metals (Co, Mn, Ni, Ce). The impregnated supports were dried at 80 °C for 24 h and calcined at 450 °C for 9 h. The specific surface area of the obtained catalysts measured with the BET isotherm method ranged from 6 to 9 m^2^/g. The cobalt-containing catalyst was also obtained in its reduced form by subjecting it to hydrogen at a temperature of 500 °C for 24 h.

Gases were analyzed using two gas chromatographs. Hydrogen, carbon monoxide, methane, and carbon dioxide were analyzed on a Chrompack CP 9002 equipped with a TCD detector and a Porapack Q column. The C_2_ hydrocarbons (acetylene, ethylene, and ethane) were analyzed on an Agilent 6890N gas chromatograph equipped with FID and TCD detectors and a ShinCarbon column. The carrier gas in both chromatographs was argon.
